# *Brucella abortus* and public health risk: Prevalence in milk sold at open markets in Cameroon

**DOI:** 10.1371/journal.pntd.0014051

**Published:** 2026-03-23

**Authors:** Pierre Gontao, Christopher G. Laine, Gaelle Kamdjo Guela, Charles Olivier Gomsu Dada, Hortense Abiba, Charles Félix Bilong Bilong, Wilfred Mbacham, Daniel G. Garcia-Gonzalez, Sonia Vection, John D. Gillece, Jeffrey T. Foster, Abel Wade, Angela M. Arenas-Gamboa

**Affiliations:** 1 National Veterinary Laboratory, Ministry of Livestock, Fisheries and Animal Industries, Yaoundé, Cameroon; 2 Department of Animal Biology and Physiology, University of Yaoundé I, Yaoundé, Cameroon; 3 Department of Veterinary Pathobiology, College of Veterinary Medicine & Biomedical Sciences, Texas A&M University, College Station, Texas, United States of America; 4 Pathogen and Microbiome Institute, Northern Arizona University, Flagstaff, Arizona, United States of America; Makerere University, UGANDA

## Abstract

Brucellosis, a neglected zoonotic disease, is endemic in many sub-Saharan African countries, including Cameroon. Recent studies have shown that *Brucella abortus* infects multiple livestock species throughout Cameroon. While the presence and risk to humans have been identified at farms and abattoirs, the bacterium has never been detected in milk intended for human consumption. In Cameroon, milk is commonly sold at open-air markets for human consumption without regulation. This study investigates the presence of *Brucella* spp. in milk sold at these markets in three regions: the Far North, North, and West. Cow milk samples and surveys were collected from 380 merchants in each region, totaling 1,140 samples and surveys. Each sample underwent iELISA, culture, real-time PCR, and next-generation sequencing (NGS). Surveys assessed the knowledge, attitudes, and practices (KAP) of milk merchants. Results indicate that *B. abortus* is not only endemic in livestock but present in milk sold for human consumption. Culture results show a countrywide positivity of 1.3% (1.1% Far North, 1.1% North, 1.8% West). Real-time PCR results indicate a 4.0% positivity rate (2.4% Far North, 5.5% North, 4.0% West). iELISA indicates 21% of the milk samples contained anti-*Brucella* antibodies, with significant regional variations (38.4% Far North, 15.5% North, 8.9% West). Finally, NGS revealed that the bacterial isolates from milk are epidemiologically linked to those obtained from animals across the region. KAP analysis shows that only 26.4% of merchants boil and 91.2% mix the milk from multiple cattle before sale. All surveyed merchants selling culture-positive milk engaged in risky behaviors, resulting in the sale of contaminated milk to approximately 720 people each week. This study highlights the risk of human brucellosis extending beyond farms and abattoirs to the general public. Future research should investigate milk consumers’ habits and prevalence to better understand brucellosis risk in Cameroon.

## Author summary

Brucellosis is a neglected disease that affects both animals and humans, particularly in sub-Saharan Africa. In Cameroon, *Brucella abortus*, a bacterium that primarily infects cattle, has been found to infect multiple livestock species, posing a risk to people working on farms and in slaughterhouses. However, this study reveals that the bacterium is also present in milk sold at open-air markets, which are a common source of food for the general public. Milk samples from 380 merchants in each of three regions were collected and assessed using serology, culture, real-time PCR, and next-generation sequencing of the isolate DNA. It was discovered that *B. abortus* is indeed present in milk intended for human consumption, and milk merchants engage in well-known risky practices, such as not boiling milk before sale. This finding is significant because it shows that the risk of brucellosis extends beyond livestock handlers to the public that drinks milk. The study highlights the need for better regulation and practices to ensure milk safety, which could help prevent the spread of this disease. Understanding and addressing these risks is crucial for improving public health and food safety in Cameroon and the region of Africa.

## Introduction

Brucellosis is a zoonotic disease caused by Gram-negative bacteria from the *Brucella* genus. Three species are particularly virulent to both animals and humans: *Brucella abortus* (cattle), *Brucella melitensis* (sheep and goats), and *Brucella suis* (swine) [1–3]. The disease is of significant concern in endemic regions globally such as large portions of Africa and Asia [1,4,5], leading to considerable direct and indirect economic losses, including those from abortion, infertility, reduced milk production, and the delivery of weak offspring [1,3]. In animals, transmission typically occurs through aerosolized particles from infected uterine secretions, fetal membranes, or aborted fetuses [3,6]. In humans, brucellosis is associated with high morbidity worldwide [4] and generally presents with non-specific, influenza-like symptoms such as fatigue, sweats, malaise, and arthritis [3,7]. If untreated, it can progress to severe, life-threatening conditions including endocarditis and neurological disorders [3,7]. Recent studies estimate about 2.1 million new human cases per year globally, with the highest risk of infection in sub-Saharan Africa [4]. Human transmission can occur via aerosolization when handling infected animal tissues, particularly during birthing, posing a significant risk to veterinarians and animal workers [1,3]. However, for the general population, transmission is mainly foodborne, typically through the consumption of unpasteurized milk and dairy products [1,3]. In many countries worldwide, milk is typically consumed in its natural or fermented form, with pasteurization being rare due to cultural beliefs that it diminishes the milk’s quality and health benefits. Additionally, practices including hand milking, communal collection in reused containers, and poor transportation and storage are common, heightening the risk of disease transmission. Cameroon, a country in West-Central Africa was recently identified as endemic for *B. abortus* [8].

This study aimed to assess the potential exposure and risk of foodborne transmission of *Brucella* through the consumption of contaminated milk purchased in open-air community markets in Cameroon, a West-Central African country lacking a national surveillance or control program that includes vaccination. The study investigated *Brucella* in milk at local markets and included: 1) bacteriological and molecular evaluation, 2) estimation of prevalence, 3) identification of risk practices associated with the sale of contaminated milk, and 4) an assessment of potential public exposure. Our goal was to help address knowledge gaps in milk products contaminated with *Brucella* and provide a clearer understanding of the public health risk not only in Cameroon, but to the broader region, with the ultimate intention of developing new interventions to help reduce the disease burden.

## Methods

### Ethics statement

This study was evaluated and approved by the: 1) Texas A&M University (TAMU), Offices of Research Compliance and Biosafety, Human Research Protection Program (HRPP) and United States Department of Defense (DOD), Office of Human Research Oversight (OHRO), Research Oversight Board (ROB) (IRB2020-0974D), and 2) United States Department of Defense (DOD), Defense Threat Reduction Agency (DTRA), Research Oversight Board (ROB) (registration number CT00008). The U.S. Army Medical Research and Development Command, Office of Research Protections (MRDC ORP), Human Research Protections Office (HRPO) registered this effort as E02473, and approved research involving human subjects. The HRPO log numbers are the following: E02473.1a (TAMU Site), E02473.1b-1 (LAVANET-West Region Site), E02473.1b-2 (LAVANET-Far North Region Site) and E02473.1b-3 (LAVANET-North Region). LANAVET Cameroon deferred to the TAMU IRB and DOD ROB. Informed consent was written.

### Sampling strategy

**Fig 1:**
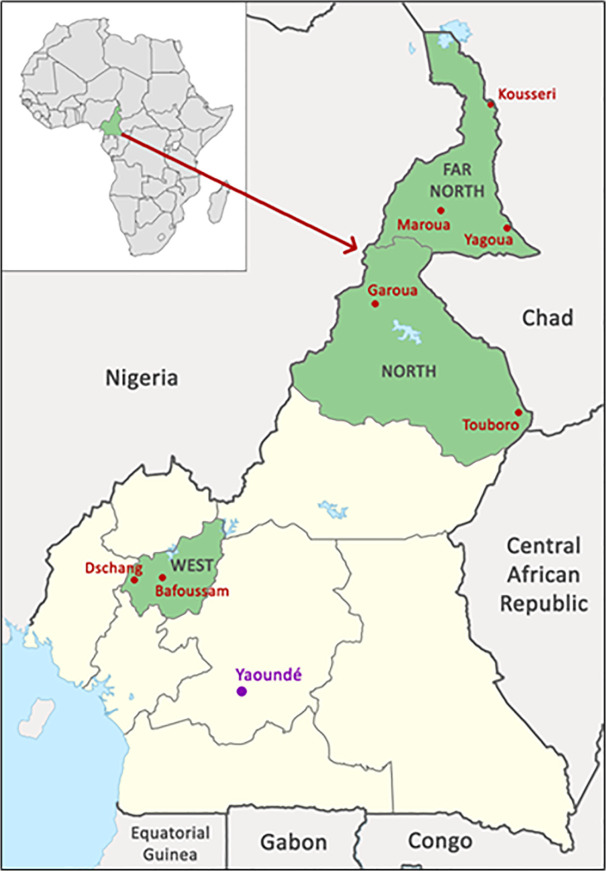
Map of Cameroon. Sampling regions (Far North, North, and West) shaded in green, along with the sample site cities (Kousseri, Maroua, Yagoua, Garoua, Touboro, Dschang, and Bafoussam) in red. The location of laboratory analysis (Yaoundé; the capitol of Cameroon) is depicted in purple.

This study was carried out in open-air community markets across three regions of Cameroon: the Far North, North, and West. In the Far North, samples were collected from the cities of Maroua, Yagoua, and Kousseri (Fig 1). In the North region, Garoua and Touboro were the sampling locations, while in the West, cities of Bafoussam, Dschang, and Foumban were included. These sites were selected based on a prior study indicating that *B. abortus* is endemic to the country, primarily affecting cattle in these three regions [8].

From February to September 2023, milk samples from randomly selected merchants were collected in each of the three regions. A total of 1,107 samples across the country were required based on limited historical data on seroprevalence in livestock, which suggested an estimated prevalence of 4.0% [12–20]. With a target precision of ± 2.0% and a 95% confidence interval (CI), a minimum of 369 milk samples were collected per region to achieve the desired statistical power. For each sample, 10 mL of fresh milk was collected into individual sterile containers and stored at -20⁰ C until testing.

### Survey

A short structured, closed-ended survey was administered to each milk merchant that provided a sample (S1 File). Electronic tablets using the Open Data Kit (ODK) Collect software were employed for answer collection and data transfer. Question topics included: demographic data, information about milk collection, pasteurization, sale, and their personal consumption practices.

### Bacterial culture and isolation

For bacteriological culture, the entire milk sample was centrifuged at 1,700 x g for 15 minutes. The skim milk was discarded, and the the cream layer and pellet were combined. This suspension was plated onto two Farrell’s agar plates, per the WOAH guidelines [2]. The plates were incubated at 37°C and bacterial growth monitored for up to 10 days. Following growth, bacterial colonies resembling the phenotype and growth pattern consistent with the *Brucella* spp. were subsequently subcultured onto Tryptic Soy Agar (TSA). DNA was extracted from suspect colonies and species identity was confirmed using real-time PCR as described in the subsequent molecular analysis section.

### DNA extraction

A quick lysis preparation for genomic DNA was performed on all bacterial colonies exhibiting *Brucella* spp. phenotype and growth, by removing a small portion of the colony using a sterile inoculation loop and mixing thoroughly in 50 μL of DNase-free sterile water. The suspensions were heated in a dry bath at 95°C for 10 minutes. Genomic DNA from confirmed *Brucella* isolates was extracted using mericon DNA Bacteria Plus Kit (Qiagen) according to the manufacturer’s instructions. Genomic DNA was also extracted directly from every milk sample using the DNeasy PowerFood Microbial Kit according to manufacturer’s instructions (Qiagen).

### Molecular analysis

DNA samples extracted from isolates and milk were analyzed using real-time PCR. DNA concentrations obtained after extraction allowed for the direct use of DNA for real-time PCR analysis. First, *IS711* was amplified and used to confirm the genus, *Brucella,* and the species were identified using species-specific real-time PCR primers (Table 1) [9]. The reaction for the species-specific real-time PCR was conducted in a 25 μL total volume, containing 5 μL of DNA, 12.5 μL of TaqMan Universal Master Mix No AmpErase UNG (Life Technologies), 6.9 μL of DNase-free water, 0.2 μL of each 20 μM forward and reverse primer (Sigma Aldrich), and 0.2 μL of 10 μM FAM Probe (Sigma Aldrich) (Table 1). Real-time PCR was performed on a CFX96 real-time PCR System (Bio-Rad) with the following conditions: initial denaturation at 95°C for 10 minutes, followed by 45 amplification cycles at 95°C for 15 seconds, and an elongation step of 1 minute at 62°C. Genomic DNA from *B. abortus*, *B. melitensis*, and *B. suis* were used as positive controls, while sterile DNA-free water served as the negative control. Results were reported as Cq values. Samples with a Cq value ≤ 40 were considered positive. The cut-off value was determined using a receiver operating characteristic (ROC) curve, with culture and PCR-positive samples as true positives (TP) and samples negative by culture and PCR as true negatives (TN).

**Table 1:** Polymerase chain reaction (PCR) primers employed in Cameroon milk prevalence study.

Target	Forward primer/reverse primer (5′ → 3′)	Probe (5′ Fluorophore → 3′ Quencher)	Species
***IS711***	GCTTGAAGCTTGCGGACAGT/ GGCCTACCGCTGCGAAT	6-FAM- AAGCCAACACCCGGCCATTATGGT-BHQ-1	*Brucella* spp.
***BMEII0466***	TCGCATCGGCAGTTTCAA/CCAGCTTTTGGCCTTTTCC	6-FAM CCTCGGCATGGCCCGCAA BHQ-1	*B. melitensis* & *B. ovis*
***BruAb2_0168***	GCACACTCACCTTCCACAACAA/CCCCGTTCTGCACCAGACT	6-FAM TGGAACGACCTTTGCAGGCGAGATC BHQ-1	*B. abortus*
***BR0952***	CCTGCAAAAAGCAGGAACCA/CCTCCGCCAGTCGTGAAA	6-FAM ATATGGCCGGCTATCCGCGTTCG BHQ-1	*B. suis* & *B. canis*

### Next-generation sequencing

Whole genome sequencing of all *Brucella*-positive DNA was performed at the Agricultural Research Council’s Biotechnology Platform (ARC-BTP) in South Africa, following the MGIEasy protocol. DNA samples were mechanically fragmented using the E220 Focused-ultrasonicator, producing fragments between 350 and 500 bp. These fragments were then selected and purified using MGIEasy DNA Clean beads. The DNA underwent end-repair and dATP tailing (ERAT) with the appropriate buffer and enzyme mix, facilitating the ligation of MGIEasy DNA adapters. After adapter ligation, the DNA products were cleaned, and each sample underwent seven cycles of PCR amplification using a Bio-Rad thermocycler. The amplified products were purified with magnetic beads and assessed for fragment size distribution using the dsDNA fluorescence kit. The amplicons were denatured and subjected to single-stranded circularization. Any uncirculated or linear DNA strands were digested with an exonuclease, resulting in a single-stranded circular DNA library.

DNA nanobeads (DNBs) were generated using rolling circle amplification (RCA) technology. The MGIEasy circularization kit (MGI, China) was used for circularized DNA, while the DNBSEQ-G400RS high-throughput sequencing kit (MGI, China) was used for DNBs. The DNA nanobeads were then loaded onto a sequencing chip for PE150 sequencing on the DNBSEQ-G400 (MGI, China).

Synthesis-by-sequencing was conducted on pooled libraries using the Illumina NovaSeq 6000 platform. Read quality was assessed with FastQC v0.11.9, and reads containing adapter sequences or low-quality data were trimmed with trimmomatic v0.39 [10]. Samples without adequate coverage for phylogenetic placement were excluded from the analyses. Sequences are available at the Sequence Read Archive (SRA) of the National Center for Biotechnology Information (NCBI), BioProject XXX [11].

### Whole genome sequencing and phylogenetic analyses

A set of whole genome assemblies representing the biogeographical diversity of *B. abortus* was downloaded from NCBI. Concomitantly, *B. abortus* SRA raw sequence data of samples from Africa were downloaded to provide regional context for the samples sequenced in this study [11]. A matrix consisting of orthologous single nucleotide polymorphisms (SNP) was constructed from the assemblies, the SRA data, and the isolates that were collected and sequenced for this study using the Northern Arizona SNP Pipeline [13]. Nucleic Acid Secondary Predictor (NASP) is a tool that wraps myriad functions, including alignment to a reference, SNP identification, and the generation of a data table with high-quality polymorphic loci. We required any given site to contain at least 10X coverage, of which 90% of the calls had to agree for any given base to be included in the final matrix. The final matrix contained 9,488 SNPs. A maximum likelihood phylogenetic tree was constructed from the matrix using IQ-TREE [12].

### Indirect enzyme-linked immunosorbent assay (iELISA)

Milk samples were tested for antibodies against smooth strains of *Brucella* using an in-house iELISA [2]. The assay was initially developed and validated in the United States, then further validated at LANAVET under local conditions using regional samples before implementation. In brief, 96-well plates were coated with 2.5 µg/well of heat-killed *B. abortus* S19 lysate in coating buffer (0.05 M carbonate buffer, pH 9.6) and incubated overnight at 4°C. The next day, plates were washed three times with PBS containing 0.05% Tween 20 (PBST) and blocked with 200 µL of 0.25% bovine serum albumin (BSA) in PBS at 37°C for 2 hours. After washing, milk diluted 1:1 was added to the wells and incubated at 37°C for 1 hour. Plates were washed again, and anti-bovine IgG conjugated to HRP (LCG Diagnostics) was added to the wells and incubated at 37°C for 1 hour. Afterward, o-Phenylenediamine dihydrochloride (OPD) peroxidase substrate (100 µL/well) was added, and the plates were incubated in the dark at 37°C for 30 minutes. Absorbance was measured at 450 nm using an ELISA plate reader. The cut-off value was determined using a ROC curve, with culture and real-time PCR positive samples as true positives (TP) and samples negative by culture and real-time PCR as true negatives (TN). All tests were conducted in triplicate, with results reported as mean optical density (OD) values for the three wells. An average absorbance ≥ the threshold was considered a positive result, while an absorbance < the threshold was classified as negative.

### Data analysis

Prevalence was estimated using binomial exact calculations with a 95% confidence interval (CI), weighted by population size. Using a two sample Z-test of proportions to determine whether populations differ significantly, positivity rates were assessed using a 95% CI and 80% power, with statistical significance defined as p ≤ 0.05. For the real-time PCR, ROC cut-off values were determined by optimizing both sensitivity and specificity, based on TP and TN samples. Similarly, for the iELISA, ROC cut-off values were established by maximizing sensitivity and specificity, using TP and TN milk samples collected from Cameroon through culture and/or real-time PCR. Data from all diagnostic tests performed on each livestock species were summarized both nationwide and by region.

## Results

Milk samples and surveys were taken from 380 milk merchants per region, for a total of 1,140, at open air community markets in the Far North (380), North (380), and West (380) regions of Cameroon (1,140). Every milk sample was subjected to culture and isolation, real-time PCR, and iELISA.

### Bacterial culture and isolation, and real-time PCR analysis

Milk samples were cultured, and *Brucella* was successfully isolated from all sample regions (Table 2). Nationwide, 1.3% of the samples tested positive by culture (Table 2). Regionally, 1.1% of samples were positive in the Far North, 1.1% in the North, and 1.8% in the West (Table 2). Real-time PCR analysis with species-specific primers (Table 1) was performed on all bacterial isolates, and all 15 isolates were identified as *B. abortus* (Table 2). Additionally, to evaluate the potential of using real-time PCR directly on the samples, DNA was extracted from the milk and tested with the assay. This method detected *Brucella* in an additional 30 samples (Table 2), all identified as *B. abortus* (Table 1). Nationwide, 4.0% of the milk samples were positive by real-time PCR, with the North exhibiting the highest rate at 5.5%, followed by the West at 4.0%, and the Far North at 2.4% (Table 2). Comparisons were made to determine if culture or real-time PCR positivity was regionally associated, but no statistically significant differences were observed.

**Table 2:** Culture, real-time PCR, and indirect enzyme-linked immunosorbent assay (iELISA) test results for milk samples.

Region	Culture: Pos/ Neg % Pos	Real-time PCR: Pos/ Neg % Pos	iELISA: Pos/ Neg % Pos
**Countrywide**	15/ 1,125 1.3%	45/ 1,095 4.0%	239/ 901 21.0%
**Far North**	4/ 376 1.1%	9/ 371 2.4%	146/ 234 38.4%
**North**	4/ 376 1.1%	21/ 359 5.5%	59/ 321 15.5%
**West**	7/ 373 1.8%	15/ 365 4.0%	34/ 346 8.9%

Number of milk samples that tested positive or negative by a particular diagnostic test, including their rate of positivity countrywide and by region of Cameroon. Pos = Positive; Neg = Negative.

### Next-generation sequencing

**Fig 2:**
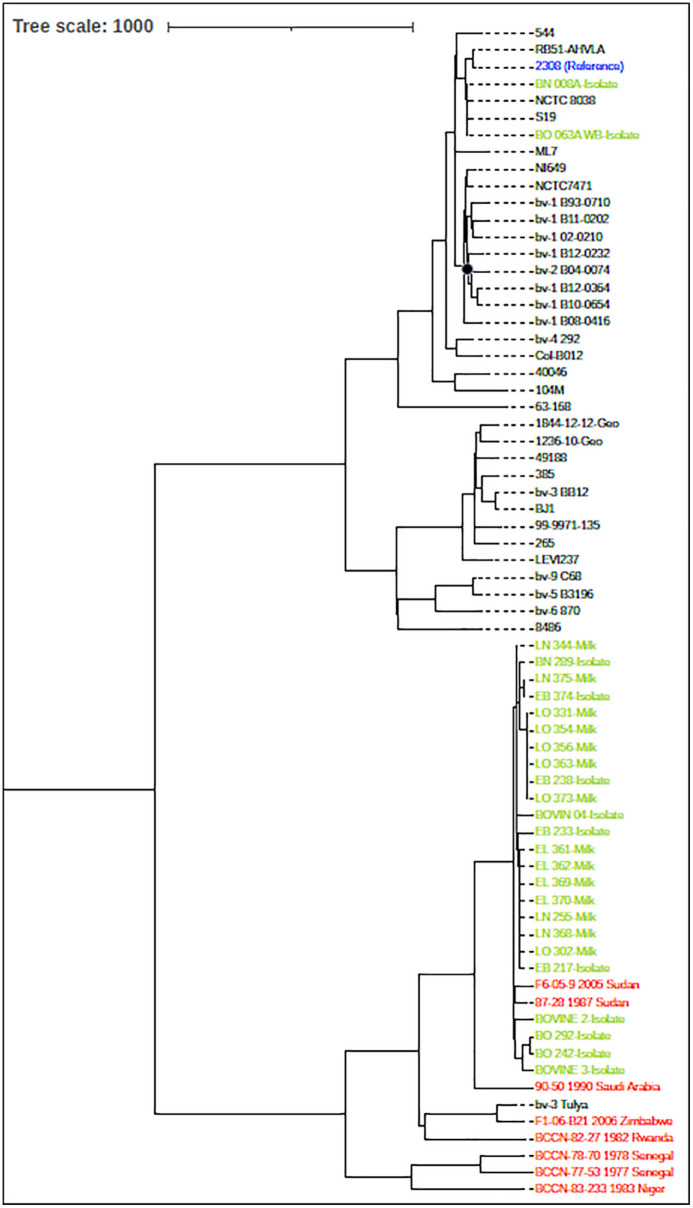
Maximum parsimony phylogenetic tree of *Brucella abortus* samples that was generated from a matrix containing 9,768 polymorphic sites. Three different sources of samples were included in the tree: whole genome assemblies (black), sequence read archive (red), and sequence data from samples collected and generated for this study (green). There was only a single branch denoted by a black circle that had a bootstrap value (1000 replicates) less than 1, which was 68. The scale bar of 1000 reflects the number of SNPs. The sequences obtained from milk samples in this study cluster with *Brucella abortus* isolates from cattle collected in the same regions. Importantly, these milk-derived sequences do not group with any vaccine strains. In the figure, green symbols represent these samples, where L = milk, B = cattle; E = Far North, N = North, and O = West.

A phylogenetic tree was generated containing the *Brucella* isolates from 14 milk samples that were sequenced and compared to the SRA and whole genome assemblies as described in the methods. Each of the strains is closely related to the biovar 3 reference strain, Tulya (Fig 2). As expected, the milk samples collected from Cameroon are more closely related to each other than the remainder of the samples within the Tulya clade that were collected outside of Cameroon.

### Serological analysis

In addition to culture and real-time PCR, the in-house iELISA was employed to determine the presence of anti-*Brucella* antibodies in milk, as it can serve as an effective tool for screening [2]. Nationwide, 21% of the milk samples tested positive by iELISA (Table 2). Specifically, milk from the Far North had a significantly higher positivity rate of 38.4%, compared to 15.5% in the North (p < 0.0005) and 8.9% in the West (p < 0.0005) (Table 2). Additionally, positivity in the North was significantly higher than in the West (8.9%, p < 0.0005) (Table 2).

### Survey analysis

A brief, structured, closed-ended survey was administered to each milk merchant who provided a sample (S1 File). Nationwide, practices related to merchants having positive culture test results were generally consistent, both across the country and by region (Table 3). Due to the limited number of culture-positive samples (n = 15), statistical power was insufficient to compare proportions between merchants selling culture-positive milk and those selling negative milk. Despite this, it was found that milk was typically sold within approximately six hours of collection and sourced from local farms (98.1%), which were on average 7.3 km from the markets (Table 3). While most milk was locally sourced, 15.0% of merchants sold milk at other markets, with merchants from the West (38.3%) selling elsewhere significantly more often than those from the Far North (3.6%, p < 0.0005) and North (2.2%, p < 0.0005) regions (Table 3). Regarding whether merchants boiled their milk before selling it, only 26.4% of all merchants answered affirmatively, with 13.3% of merchants selling culture-positive milk also responding affirmatively to boiling their milk (Table 3). Notably, merchants from the North (49.2%) boiled their milk significantly more often than those from the West (19.5%, p < 0.0005) and Far North (10.6%, p < 0.0005) regions (Table 3). Nearly all merchants were milking multiple cattle (an average of 10), with over 90% combining milk into a single container before sale (Table 3). Furthermore, 53.8% of merchants selling culture-positive milk mixed their milk with milk from animals they did not own (Table 3). Merchants from the Far North and West regions (99.5% and 99.7%, respectively) combined milk at significantly higher rates than those from the North (73.1%, p < 0.0005) (Table 3). It is noteworthy that 100% of merchants selling culture-positive milk and 98.4% of all surveyed merchants engaged in at least one risk factor for selling contaminated milk (i.e., not boiling milk, mixing milk from multiple cattle into a single container, and combining milk from animals they do not own). Additionally, merchants selling the positive milk sold it approximately four days per week to an average of 12 people per day, with 86.7% providing it to their families (Table 3).

**Table 3:** Milk merchant survey results, countrywide and by region of Cameroon.

		Countrywide		Regions		
Survey Questions	Units	Culture Positive Samples	All Samples	All Far North Samples	All North Samples	All West Samples
**How long ago did you collect this milk?**	Hours	4.3 avg (SD = 2.7)	5.8 avg (SD = 6.0)	3.7 avg (SD = 3.1)	7.9 avg (SD = 4.5)	5.9 avg (SD = 8.3)
**Does this milk come from animals living in this area?**	Yes	92.9%	98.1%	99.5%	93.4%	100.0%
**How far away?**	Km	4.5 avg (SD = 2.3)	7.3 avg (SD = 7.1)	4.0 avg (SD = 1.4)	18.0 avg (SD = 7.5)	3.8 avg (SD = 1.3)
**Do you sell this milk anywhere else?**	Yes	13.3%	15.0%	3.6%	2.2%	38.3%
Do you boil the milk before you sell it? *	Yes	13.3%	26.4%	10.6%	49.2%	19.5%
Does the milk that you sell come from one or multiple animals? *	Multiple	100%	98.2%	100.0%	92.4%	99.7%
**How many cows are you currently milking?**	Cows	7.9 avg (SD = 3.0)	10.0 avg (SD = 4.8)	7.9 avg (SD = 2.9)	15.7 avg (SD = 6.2)	8.5 avg (SD = 2.4)
Do you mix this milk into a container before selling it? *	Yes	86.7%	91.2%	99.5%	73.1%	99.7%
Do you combine any milk from animals that you do not own? *	Yes	53.8%	41.2%	27.9%	79.8%	28.0%
**How many days per week do you sell milk?**	Days	3.9 avg (SD = 1.3)	3.6 avg (SD = 1.8)	3.7 avg (SD = 1.9)	3.9 avg (SD = 2.1)	3.2 avg (SD = 1.3)
**Approximately how many people per day do you sell milk to?**	People	12.4 avg (SD = 6.0)	16.0 avg (SD = 7.0)	17.1 avg (SD 8.0)	12.9 avg (SD = 6.1)	17.4 avg (SD = 5.1)
**Do you or your family drink milk from the animals that this milk comes from?**	Yes	86.7%	82.1%	94.3%	90.8%	60.9%
Merchants practicing at least 1 risk factor *	Yes	100%	98.4%	100%	95.30%	100%

Survey results from 1,140 milk merchants at open air community markets in the Far North (380), North (380), and West (380) regions of Cameroon. * Known Risk Factors; avg = Average; SD = Standard Deviation; Km = kilometers.

## Discussion

*Brucella* has long been recognized as endemic across Africa, making the region the highest-risk area for human brucellosis globally [4]. However, despite this recognition, the full extent and impact of the disease on humans remain poorly understood, particularly in West Africa [13]. Furthermore, dairy products remain vital sources of high-quality protein and essential nutrients including calcium [14] as global population growth increases the demand for milk [15,16]. Dairy production also plays a critical role in supporting nutrition and local economies by creating income opportunities that positively impact public health [17]. This study focused on a unique opportunity of determining whether milk sold to the general public throughout Cameroon could pose a significant public health risk for acquiring brucellosis. While previous research conducted in the country has aimed to address knowledge gaps in livestock prevalence, to our knowledge, no studies have attempted to isolate, characterize, and establish epidemiological links between bacteria found in milk sold in local markets and those present in multiple livestock species. Additionally, no studies have assessed the public health risk factors associated with the open sale of contaminated milk to the general public.

As in most African countries, milk in Cameroon is primarily sourced from local markets, where small-scale farms contribute to the supply. Throughout the country, milk for home consumption is typically obtained from two main sources: 1) local open-air community markets, where small-scale farms sell unpasteurized milk (the primary source), and 2) supermarkets, which offer commercially produced pasteurized milk. Consequently, local markets were chosen as sampling sites. To accurately estimate the proportion of suppliers providing contaminated milk in the selected regions and to establish epidemiological links between bacteria in milk sold in local markets and those present in multiple livestock species, random samples of milk were collected from merchants at these sites. The samples were then analyzed using bacteriological and molecular testing, along with next-generation sequencing.

Culture and real-time PCR analysis of milk samples from over 1,140 merchants demonstrated that *B. abortus* is present in milk sold for human consumption across all sampled regions. In addition, to determine the presence of antibodies against the disease in milk iELISA was employed [18] which can serve as a tool for screening [2]. Milk that was positive for *B. abortus* was detected nationwide; however, when analyzing Cameroon’s regions individually, the highest concentration of *Brucella* culture, real-time PCR, and iELISA positive milk was found in the Far North (Table 2), with only the iELISA findings reaching statistical significance. Furthermore, all isolates were epidemiologically linked to previously identified *B. abortus* strains from cattle in each sampled region [8]. Given the unregulated movement of livestock, genomic analysis suggests that the endemic *B. abortus* strain in Cameroon may have originated from the Sudanese region of eastern sub-Saharan Africa, as most *B. abortus* genotypes closely matched two strains from Sudan, isolated in 1987 and 2005. These isolates belong to the Tulya clade, named after a reference strain collected from a patient in Uganda in 1958 [19]. Notably, the strains isolated in this study are closely related to *B. abortus* biovar 3 (Bv3), which, along with Bv6, is commonly associated with African lineages [20]. This finding highlights the widespread presence of African endemic strains in Cameroon [20], as has been identified in other countries in West Africa [21]. Additionally, due to porous borders and active international trade routes, similar transmission patterns are likely in neighboring countries. Expanding future research to include these countries would provide a more comprehensive understanding of *Brucella* public health impact in the region.

After administering a brief, structured, closed-ended survey to each milk merchant that provided a sample, this study found that 100% of merchants selling culture-positive milk and 98.4% of all surveyed merchants were involved in at least one risk factor for selling contaminated milk (e.g., not boiling milk, mixing milk from multiple cattle in a single container, and combining milk from animals they do not own). Merchants selling positive milk typically did so approximately four days per week, serving an average of 12 people per day. This amounts to a potential of 720 sales of milk contaminated with *B. abortus* among the sample population, highlighting a significant public health risk as well as the need for education and intervention. Additionally, with a 1.3% prevalence of culture-positive milk, an individual randomly choosing a vendor would have roughly a 1 in 77 chance of purchasing contaminated milk. Over a lifetime of milk consumption, this risk of exposure and infection becomes notably significant.

One factor not captured in our survey but widely recognized in similar settings, is the lack of refrigeration or cold chain during milk collection, transport, and sale. Although milk is typically sold within hours of collection, it is often sold at ambient temperatures exceeding well over 30°C in the Far North and North regions. Implementing cold chain infrastructure to improve milk safety remains a major challenge in resource-limited settings. Drinking or eating products made from raw milk can expose people to many pathogens including *Brucella* and lead to serious health risks, especially for certain vulnerable populations [22]. Pasteurization is essential for milk safety, killing harmful pathogens and is the most effective and straightforward method to prevent transmission of this disease to humans while enjoying milk’s nutritional benefits [22]. This study demonstrates that the *B. abortus* found in milk is epidemiologically linked and closely related to the strain present in multiple livestock species, primarily cattle. Therefore, controlling the disease in cattle is vital to enhance food safety and prevent related foodborne illnesses. Since handling infected animals and consuming raw dairy products are major risk factors for human brucellosis, future research should focus on both farmers and dairy consumers to better understand the extent of the human brucellosis burden in the country.

Current knowledge of human brucellosis in Cameroon is extremely limited [23]. Available reports suggest that the disease is likely underdiagnosed due to weak surveillance infrastructure and the absence of pathognomonic clinical signs, which often leads to misclassification as other febrile illnesses [23–25]. Official incidence data for humans are scarce, and national reporting systems do not routinely capture brucellosis cases [23,24]. Similarly, estimates of bovine brucellosis prevalence vary by region, but comprehensive national-level data remain fragmented [23]. This lack of reliable information underscores the importance of our findings for contextualizing the potential public health risk and highlights the need for integrated surveillance systems linking animal and human health sectors.

Specifically, it is important to assess purchaser boiling practices to investigate the direct risk to consumers. Additionally, control strategies should target the national and international cattle supply chain to prevent *B. abortus* contamination in milk and mitigate the associated public health risks. To our knowledge, this is the first study of its kind in the region, and its findings lay a critical foundation for developing effective public health surveillance, control, and educational policies in Cameroon, West Africa, and potentially across the continent.

## Supporting information

S1 File Milk Merchant Survey Form. (PDF)

S2 File Milk Merchant Survey Data. (XLSX)

S3 File Milk Diagnostic Data. (XLSX)
