# Supplementary material for: Brucella abortus and public health risk: Prevalence in milk sold at open markets in Cameroon
Source: PLoS Negl Trop Dis. 2026 Mar 23;20(3):e0014051. doi: 10.1371/journal.pntd.0014051 (PMC13008051; doi:10.1371/journal.pntd.0014051)
Supplement: S1 File — (PDF) [file pntd.0014051.s001.pdf]

Milk Survey Form

<u>This Form is Only to be Used if the Tablet is not Functional</u>

Milk Survey

Surveyor Name ____

Date (Year/Month/Day) of Sample Collection ____

Time (24-hour scale) of Sample Collection ____

Name of the Region ____

Name of the City ____

Enter the first and last name of the person being surveyed:

____

Milk sample IDs taken from this individual (ID Numbers):

____

Has the person given consent to be surveyed? (Check One):

(They must give consent)

☐ Yes

☐ No

1. How long ago did you collect this milk? (Hours, Days) ______________________________

2. Do you boil the milk before you sell it?
   - ☐ Yes
   - ☐ No (Skip to Question #5)
   - ☐ No Answer (Skip to Question #5)

3. Do you heat the milk until it bubbles?
   - ☐ Yes
   - ☐ No (Skip to Question #5)
   - ☐ No Answer (Skip to Question #5)

4. How long did you heat this milk while it bubbled? (Number of Minutes) ________

5. Does the milk that you sell come from one ore multiple animals?
   - ☐ One (Skip to Question #9)
   - ☐ Multiple
   - ☐ No Answer (Skip to Question #9)

6. How many cows are you currently milking? ________

7. Do you mix this milk into a container before selling it?
   - ☐ Yes
   - ☐ No (Skip to Question #9)
   - ☐ No Answer (Skip to Question #9)

8. Do you combine any milk from animals that you do not own?
   - ☐ Yes
   - ☐ No
   - ☐ No Answer

9. Does this milk come from animals living in this this area?
   - ☐ Yes
   - ☐ No (Skip to Question #11)
   - ☐ No Answer (Skip to Question #12)

10. How far away? (Kilometers) ________ (Skip to Question #12)

11. Where are the animals that this milk comes from? (Region, Division, Sub-division)

    ____

12. Do you sell this milk anywhere else?
- ☐ Yes
- ☐ No (Skip to Question #14)
- ☐ No Answer (Skip to Question #14)

13. Where do you sell this milk? (Region, Division, Sub-division) ___________________
____

14. How many days per week do you sell milk? ________

15. Approximately how many people per day do you sell milk to? ________

16. Do you or your family drink milk from the animals that this milk comes from?
- ☐ Yes
- ☐ No
- ☐ No Answer

17. Do you ever sell products made with the milk from the same animals? (Yes / No)
- ☐ Yes
- ☐ No (End Survey)
- ☐ No Answer (End Survey)

18. Do you boil the milk before making these products?
- ☐ Yes
- ☐ No
- ☐ No Answer

Formulaire d'enquête : Lait

Ce formulaire ne doit être utilisé que si la tablette n'est pas fonctionnelle

Enquête sur le lait

Nom de l'enquêteur ____

Date (Année/Mois/Jour) du prélèvement d'échantillons _______________________________

Heure (format 24 heures) du prélèvement d'échantillons ______________________________

Nom de la Région ____

Nom de la Ville ____

Noter le prénom et le nom de famille de la personne interrogée :

____

Numéro d'identification des échantillons de lait récupérés auprès de cette personne (numéro ID) :

____

La personne a-t-elle consenti à être interrogée ? (Cochez une case):

(Ils doivent donner leur consentement)

☐ Oui

☐ Non

1. Depuis combien de temps avez-vous récupéré ce lait ? (Heures, Jours) ______________________

2. Faites-vous bouillir le lait avant de le vendre ?
   - ☐ Oui
   - ☐ Non (Passer à la Question #5)
   - ☐ Pas de réponse (Passer à la Question #5)

3. Faites-vous chauffer le lait jusqu'à ce que des bulles se forment ?
   - ☐ Oui
   - ☐ Non (Passer à la Question #5)

4. Combien de temps avez-vous chauffé le lait pendant qu'il y avait des bulles ? (Nombre de minutes) ____

5. Est-ce que ce lait provient d'un seul animal ou plusieurs ?
   - ☐ Un (Passer à la Question #9)
   - ☐ Plusieurs
   - ☐ Pas de réponse (Passer à la Question #9)

6. Combien de vaches trayez-vous en ce moment ? ________

7. Est-ce que vous mélangez ce lait dans un contenant avant de le vendre ?
   - ☐ Oui
   - ☐ Non (Passer à la Question #9)
   - ☐ Pas de réponse (Passer à la Question #9)

8. Combinez-vous ce lait avec celui d'animaux qui ne vous appartiennent pas ?
   - ☐ Oui
   - ☐ Non
   - ☐ Pas de réponse

9. Est-ce que ce lait provient d'animaux vivant dans la région ?
   - ☐ Oui
   - ☐ Non (Passer à la Question #11)
   - ☐ Pas de réponse (Passer à la Question #12)

10. À quelle distance ? (Nombre de kilomètres) ________ (Passer à la Question #12)

11. Où sont les animaux qui produisent ce lait ? (Région, Division, Sous-division)
    ____

12. Vendez-vous ce lait ailleurs ?
 - ☐ Oui
 - ☐ Non (Passer à la Question #14)
 - ☐ Pas de réponse (Passer à la Question #14)

13. Où vendez-vous ce lait ? (Région, Division, Sous-division) ______________________

____

14. Combien de jours par semaine vendez-vous du lait ? ________

15. À combien de personnes (environ) par jour vendez-vous du lait ? ________

16. Est-ce que vous ou des membres de votre famille buvez le lait de ces animaux ?
 - ☐ Oui
 - ☐ Non
 - ☐ Pas de réponse

17. Est-ce que vous vendez des produits faits à partir du lait de ces animaux ?
 - ☐ Oui
 - ☐ Non (Terminer l'enquête)
 - ☐ Pas de réponse (Terminer l'enquête)

18. Faites-vous bouillir le lait avant de préparer ces produits ?
 - ☐ Oui
 - ☐ Non
 - ☐ Pas de réponse
